# Analysis of Participant Reactivity in Dyads Performing a Videotaped Conflict-Management Task

**DOI:** 10.5402/2011/596820

**Published:** 2011-06-01

**Authors:** Yulia Y. Semeniuk, Susan K. Riesch

**Affiliations:** School of Nursing, University of Wisconsin-Madison, 600 Highland Avenue, Madison, WI 53792, USA

## Abstract

Videotaping is used frequently in nursing research. A threat to the validity of videotaping is participant reactivity, that is, being recorded by a camera may influence the behavior of interest. This paper's purpose is to report how youth ages 10 to 14 years old and their parent viewed participation in a videotaped conflict-management task. Five dyads, who were part of a randomized clinical trial testing an intervention to promote parent-child communication, participated in a structured interview. All parents were mothers. Youth were eighth graders. Three were boys and two were girls. Findings indicated that (a) dyads felt that the videotaped interaction had a progression of feeling unnatural in the beginning to feeling natural toward the end, (b) dyads found it relatively easy to choose a topic of discussion, and (c) dyads felt that the discussions were meaningful. Based on these data, recommendations for researchers to reduce participant reactivity are provided.

## 1. Introduction

As children develop into adolescents, conflict with parents increases. The ability to negotiate and resolve conflict is an important element of family functioning and parent-child communication [1]. Unmanaged conflicts potentially have negative effects on the parent and child relationship [2] and youth health and development [3]. Therefore, it is important for researchers to examine and practitioners to apply strategies to assist parents and youth to acquire skills to manage conflicts. 

Universal prevention and intervention programs that target adults and youth at the transition to adolescence have been developed and disseminated. The Strengthening Families Program (SFP) 10–14 is one example. Guided by the Biopsychosocial Vulnerability Model [4] and empirically based family risk and protective factors [5], SFP 10–14 is a Substance Abuse and Mental Health Services Administration (SAMHSA) model program. A model program is one that has undergone scientific review by the National Registry of Evidence-based Programs and Practices (NREPP) (http://www.nrepp.samhsa.gov/ accessed 5 July 2010). The NREPP evaluation of the program includes an analysis of the reliability, validity, intervention fidelity, missing data and attrition, potential confounding variables, and appropriateness of analysis. NREPP criteria also address the program readiness for dissemination including an evaluation of the implementation materials, quality of the training and support resources, and quality assurance procedures. 

 The SFP 10–14 focuses on general family strengthening techniques, such as discipline, family management, communication, and stress reduction. One potential outcome of SFP 10–14 is improved parent and child conflict-management skill. In a study to examine the efficacy of SFP 10–14 for parent and youth conflict-management ability, we used a survey and macrolevel coding system to assess conflict-management ability. Parent and youth chose to discuss a recurring “hot” issue, that is, an issue of regular and exuberant disagreement between a parent and a youth from a list of 21 issue items [6]. The dyads were instructed to try to come to a resolution of the chosen hot issue while being videotaped for ten minutes. This videotaped resolution is referred to as a conflict-management task. Data collectors were not in the room while the dyads were performing the task of resolving a conflict in front of the camera. The video tapes were coded at the Iowa State University Institute for Social and Behavioral Research using the Iowa Family Interaction Rating Scale (IFIRS) [7]. The coding provided scores for quantification of conflict-management ability. 

Contrary to expectations, no significant improvement in conflict-management skill was found after program participation among the sample of 34 parents and youth (age M = 11, SD = 1.04). In other words, parent youth dyads who participated in SFP 10–14 did not demonstrate a difference in their conflict-management skill when compared with those in the control group [8]. It is possible that the experience of being videotaped was a factor influencing the dyad's ability to interact naturally. Being recorded by a video camera may influence the behavior of interest, a concept entitled participant reactivity [9].

Rather than petitioning the University's Institutional Review Board to reopen a protocol from 2002–2005, we opted to conduct interviews with families who were participating in another ongoing family intervention study using the identical videotaping procedures. The ongoing study is a National Institutes of Health-(NIH-) funded randomized clinical trial of a family communication intervention. It was thought that having an immediate recollection of the experience was more valid than asking original study participants to remember the experience of the videotaping from five years ago.

Our purpose was to assess the extent to which the videotaping process may have contributed to the study outcome thus, to examine participant reactivity as the threat to the validity of videotaping. The purpose of this paper is to examine how youth ageing 10 to 14 years old and their parents viewed participation in a videotaped conflict-management task. Specifically, the researchers sought to determine (a) how natural the interaction was perceived, (b) how difficult it was for the dyad to choose a topic, and (c) if the discussion was meaningful. 

## 2. Literature Review

Direct observation is a fundamental component of assessment of interaction in social and behavioral sciences [10, 11]. The use of video technology to capture behavior is prevalent in nursing research. Three primary uses of video technology in nursing research are documented: (a) a means of monitoring quality assurance standards and intervention fidelity; (b) a method of delivering an intervention, and (c) a method to collect research data [9]. In research, videotaping has been used to study various phenomena. For example, it has been used to study expressive behavior in persons with Parkinson's disease [12], provision of nursing care to elderly individuals with dementia [13, 14], humor in nurse/client interactions [15], parent-child/infant interaction [16, 17], and multidisciplinary teamwork in relation to discharge planning [18]. 

Videotaping may be used to enhance validity of measurement. In concert with survey methods, videotaping and coding for interaction constitutes data triangulation. To improve the validity of concept and variable measurement, such as parent and youth conflict-management skill, the use of two methods of measurement is recommended [19]. 

Researchers need to be aware of advantages and limitations of the videotaping method. Density and permanence are two principal advantages of videotaping [20]. Density means that videotaping of the data captures interaction from various angles. Notes from human observation are likely to miss certain aspects of interactions. In other words, videotaped recordings capture whole interactions compared to human observations. Permanence means that data remain unchanged for an unlimited amount of time. In addition, videotaping provides control of observer fatigue (being tired) or drift (unintentionally going off on a tangent). It further allows events to be repeated in a variety of ways (e.g., in real time, slow motion, forward, or backward) to capture verbal and nonverbal behaviors, and to analyze the same data in different ways [18, 21, 22]. 

Videotaping has limitations that affect validity, such as consistent quality of procedures used and participant reactivity to knowledge of being videotaped [9]. Because we sought to assess how videotaping a task may have affected the study outcomes, this paper will be limited to participant reactivity. 

People may change their behavior when they know they are being videotaped. Thus, the act of being recorded by a video camera may influence the behavior of interest. This phenomenon is known as participant reactivity or reflexivity to awareness of being observed [23]. Baum at al. [24] recommended the effect of reactivity be examined by asking participants whether they perceived themselves as having changed their behavior across experimental conditions. 

Taken together, the advantages and limitations of videotaping as a research data collection method reflected in the literature and the lack of an expected statistically significant change in conflict-management skill as a result of the SFP 10–14 intervention, led to our further scrutiny of the parent and youth experience with videotaping. The concept we sought to understand how further was participant reactivity. 

## 3. Method

### 3.1. Sample

From a sample of parent youth dyads who were completing their sixth and final interview as part of the randomized clinical trial testing an intervention to promote parent-child communication, five dyads were approached and agreed to an additional interview to describe their participation in the videotaped tasks. Youth were eighth graders, three were boys and two were girls. All the parents were mothers. Please see Table 1 for a summary of the social characteristics. 

### 3.2. Instrument

Structured interview questions were developed to elicit the responses of the parent and youth. There were three broad questions with specific probes. Please see Table 2 for details of the interview questions and probes. 

### 3.3. Procedure

Upon approval by the University's Institutional Review Board, five dyads who were scheduled for a sixth in-home data collection visit agreed to respond to questions about their experience with the videotaped conflict-management task. After the interview of the fifth dyad, no new ideas emerged. Data collection took place during a five-month period between January and May of 2009. 

The parent and youth separately responded to the interview questions in order to prevent influencing one another. Each interview took approximately 15 minutes. The interview was audiotaped and transcribed. Each parent and each youth received a $25 US gift certificate to a local bookstore as a token of gratitude for sharing their experiences.

We conducted a conceptual content analysis, examining the interviews for words and phrases that described the videotaping experience [19]. The transcriptions were read at least twice by a research team of faculty and graduate and undergraduate students. The first reading was to gain an overall idea of the interview content. The second reading was to analyze the content for themes, commonalities, and differences. A summary table of the ideas conveyed was assembled with representative quotations supporting the ideas. 

## 4. Results

Youth and parents were diligent about their participation in the videotaped tasks, taking the task of trying to resolve a conflict while being videotaped more seriously as their participation in the study progressed. Dyads were consistent in their responses. The major ideas expressed by the dyads were that the interaction during the videotaping sessions became more natural with each occurrence; choosing a topic to discuss was not difficult; and the relationship between parent and youth was not perceived to have been affected or changed, despite the discussion being meaningful. 

### 4.1. Naturalness

Four of the five dyads felt that the interaction while being videotaped was either natural or had a progression of feeling unnatural at the first episode to feeling more natural toward the 6th episode. Here are examples of how mothers expressed the naturalness progression:

“At the beginning it did feel unnatural. Our first 1 or 2 conversations were fairly unnatural. But, I think over time we tended to forget that the camera was there. So I think there was definitely a change over time;”
“The first time I think we were a little bit nervous, but then after [we] did it a few times, it was just like okay.”

The youth perceived the videotaping experience in the following way:

“It was really natural for me,”
“First time I was nervous and didn't really know what to say. And we finished really early. This time, we had lots to say. That's probably just because I'm older, but also, because I'm more comfortable with the camera.”

There was an exception to the naturalness progression. One dyad expressed that it did not feel natural for them to talk about the issues in front of the camera. They felt that the topic was forced. The dyad was very consistent with one another in responding to the interview questions. This was what the mother said: “It was generally unnatural. We weren't planning to have to talk about certain subject, then we were asked to do it without having it naturally come up. So, it's not something that we do anyway; we don't suddenly pick a topic to have a discussion about. So that part was unnatural” and “if arguments come up and they're more heated. I'm going to be more emotional than shown on that camera; less controlled, probably.” 

This was what the youth said: “I think it was kind of fake. It wasn't really like an actual conversation I would have had. It didn't get heated like it would if it wasn't being videotaped.” “In the beginning you just really felt forced and didn't really feel like a real conversation,” and “People can actually see that its real-life stuff, like you guys can watch it.”

In summary, four out of five dyads found that the videotaping process was either natural or had a progression of feeling unnatural in the beginning to feeling more natural toward the end. 

### 4.2. Ease of Choosing a Topic

The dyads found it easy to choose a topic. This finding was substantiated by the following quotes typical from the mothers:

“The topics were pretty good…we always came up with something to talk about…there was always…an issue…on the list that was…pretty much what…we needed to talk about,”
“Sometimes I think it what was difficult for us was choosing just…one topic. Because sometimes we have…multiple things that we could talk about that we're going through…so I think it was difficult for us to agree on a topic, and just one topic,”
“It was easy…we both focused on the same thing,” and “it really has not been that hard choosing a topic…we always come to a subject that…we have been discussing anyway, so it hasn't been difficult choosing it.”

Youth also expressed the ease of choosing the topic in the following quotes:

“It really hasn't been that hard choosing a topic,” “not very difficult. I don't know…because it was like just the main stuff, and like it was stuff that we actually go through, so it was just like very easy to pick,”
“At first it was really like hard to choose the topic because we didn't have as much to say. But now I knew exactly what I wanted to talk about, because I had anticipated talking about it,”
“The topics were pretty good…we always came up with something to talk about. Like there was always…an issue on the list that was…pretty much what we needed to talk about.”

In summary, all of the dyads found it easy to choose a topic since most of the time they had issues that they were discussing anyway or anticipated to want to talk about. One mother expressed some difficulty with narrowing the topic to just one. 

### 4.3. Meaningfulness of Discussion

The discussion was described as meaningful, but it did not necessarily affect the dyads feelings about their relationship. The following quotes from the mothers support this finding:

“[The discussion was] very meaningful to us,” “it was actually the result of one of these videotapings that we sort of changed a rule, switched something that we've been doing,”
“[The discussion] was very nice. I didn't quite problem-solve, but if there's ever a conflict I could use that as a tool, set the oven-timer for 8 minutes, and say we've got 8 minutes to sit and talk, and work this out,” and
“It gave us the opportunity to actually have to talk about stuff. Moving closer?…perhaps…that's true, because we talked about homework. I think when we don't know what's going on, it definitely pulls you apart; when you find out what's going on, you can use that opportunity to be more involved, and so you're closer in that sense. But you can blow it though by yelling and putting the kid down, and not discussing it, but making it real negative rather than creating that closeness.”

Quotes from youth about the meaningfulness of the discussion include:

“Well, not really closer but it came a lot easier because we actually got it [the issue] out there,”
“We're talking about [topic we chose] a lot. Mother even asked like other people in my family advice about it. So it's like a real topic,”
“I liked the opportunity to be able to talk with my mom about the problems,” and
“Yeah [topic was meaningful]. I think so, because some of these issues are the only issues we have, so if it helped us resolve an issue then that would help our family.”

In summary, the dyads found the discussions meaningful and liked the opportunity to talk about the issues; however, the dyads did not find that the discussions brought them closer to one another. 

### 4.4. Additional Comments and Suggestions

There were a few suggestions made by the participants regarding the videotaping and videotaped task. They suggested allowing more time for the videotaping task, specifically, “not as much time in the [beginning], and then more time in the end.” It was suggested that the task incorporate a practice session or a “dry run”. Another suggestion was having a moderator to provide tips, timing, and structure. Finally, it was suggested that if it were possible to have the same person do all six home visits with the dyad and conduct the videotaping procedures. 

## 5. Discussion

Nurse researchers incorporate videotaped tasks as measures of processes and outcomes. How does videotaping of a task contribute to the study outcomes? With each occurrence of being videotaped over six waves of interviews, the participants took the task more seriously and reported it felt increasingly natural. It was easier each time to choose a topic with some participants anticipating what they would discuss prior to the data collection period. The task was considered meaningful but it was not perceived by the dyads to have influenced their relationship.

Our findings are consistent with the literature on participant reactivity. The reactivity diminished over time as participants became acclimated to the presence of the video camera [20]. Our videotaped tasks were conducted over six waves. In future studies it would be important to investigate when participants start feeling natural while being videotaped to assist researchers in the timing of potential measures to decrease participant reactivity. 

### 5.1. Limitations

This study had three limitations. First, the dyads whose conflict-management skill was found to not change significantly as a result of intervention are not the same dyads who provided feedback on the experience of videotaping. However, the dyads did undergo the identical videotaping procedures as dyads in the SFP 10–14 study.

Second, the youth who participated in this interview were two years older than the youth who participated in the SFP 10–14 study. It is arguable that older youth may be more confident in discussing issues with their parents. However, the issues may arise more frequently and the tenor of those issues may become “hotter” than in the younger youth. 

Third, study sample size is small and the method of recruitment was not random. Five parent youth dyads participated. The method of choosing participants was inclusion of dyads whose sixth wave data collection task was scheduled at the time of the study. However, data were collected across a five-month period and were consistent and similar across dyads, with the exception of the one dyad in their response on naturalness. 

### 5.2. Recommendations

Based on our experience and the findings from these interviews, we make several suggestions to minimize participant reactivity. Researchers may conduct a practice session of the videotaped task [25, 26]. This procedure would allow the dyad to view their “videotaped selves” prior to the actual event. As a study team, our experience led us to believe that parent and youth were comfortable with videotaping because of its ubiquitous nature in society. The study results have shown that it is not necessarily true.

The procedure might be improved by starting with shorter episodes initially and adding more time in subsequent episodes. As the dyads became more comfortable, the time for the task was perceived to be short. 

Three waves of data collection were conducted in the SFP 10–14 study over a period of less than a year. This length of time may not be enough to capture the change in the conflict-management task. Thus, it is recommended that researchers follow the participants for a longer period of time that, in turn, may lead to anticipating the task and approaching it naturally. The current RCT spanned three years and six waves. 

Researchers need to routinely reiterate the privacy of the videotapes with participants at each episode. Though the videotaping procedure was described in detail in the consent form, and one youth articulated his belief that the study team would view the tape and perhaps pass judgment. The process described in the consent and assent procedures and forms indicated that nothing about the videotapes was disclosed to persons outside the team and that only the process of conflict management was of interest, not particular behaviors. Data collectors viewed the videotape only for evidence of harmful behaviors. The videotapes were sent for analysis to a third party at the Iowa State University Institute for Social and Behavioral Research where no one could identify the dyads. A regular reminder may reduce this threat to validity.

If researchers are limited to a one-session videotaping task, it is recommended that the first few minutes of the videotape be considered “pilot data.” The first few minutes in particular may be subject to participant reactivity. Analyzing the latter part of the videotape data would likely reduce the influence of participant reactivity [25, 27].

The Dyadic Assessment Intervention Model [28] is a statistical method increasingly used to analyze the dyadic quantitative data collected longitudinally. Researchers may want to consider using the first videotapes as a covariate or record two episodes for a baseline measure with the first episode used as a practice video. 

## 6. Conclusion

In conclusion, we think that the videotaping experience contributed to the validity and the measurement of the outcome variable of conflict-management skill. Videotapes during the first waves may not have been natural; dyads may not have felt free to be themselves and openly discuss the problem. In subsequent tasks, as dyads came to feel natural, it would follow that the discussion would be a valid indicator of their conflict-management ability. Thus, despite the ubiquitous nature of being videotaped, it remains a good practice to habituate the participants to the videotaping. 

##  Funding

This paper received funding from 2008-2009 Eckburg Fund Research Award from the School of Nursing, University of Wisconsin and National Institute of Health (NIH) grant no. 5R01NR007894-05, National Institute of Nursing Research. 

## Figures and Tables

**Table 1:** Description of the sample (*n* = 5).

	Dyad 1	Dyad 2	Dyad 3	Dyad 4	Dyad 5
Mother age	43	31	52	45	48
Youth age	13	13	13	13	13
Youth grade	8	8	8	8	8
Youth gender	Girl	Girl	Girl	Boy	Boy
Mother income	$40–49K	$10–19K	$50–74K	$75–99K	$50–74K
Mother education level	Some college	Some college	College graduate	College graduate	College graduate
Race	White	African American	White	White	White

**Table 2:** Interview questions with probes.

Broad interview questions	Specific probes
From the first visit to now, you were videotaped. Some people have said that it was unnatural or they were not comfortable. Others did not have any problems discussing issues while being videotaped and forgot they were being videotaped. Could you think back to when you were first videotaped to now and tell me what it was like for you?	(1) Did it feel natural/real to be videotaped? Please tell me more about that
	(2) Do you think you were yourself?
	(3) To what extent was your discussion a real dialogue?
	(4) Was there anything you liked or did not like about videotaping? Please tell me more about that

How difficult was it to choose a topic?	(1) How difficult was it to choose a topic? Please tell me more about that
	(2) How helpful was the checklist?

Was the discussion meaningful?	(1) Was the topic meaningful to your family?
	(2) Might it have moved your family closer or affected your relationship?

Are there any other comments they would make about their videotaping experience?	
